# Biochemical characterization of mulberry (*Morus* spp.) genotypes from the Türkiye (Hizan, Bitlis): A comprehensive analysis of fruit properties and bioactive compounds

**DOI:** 10.1002/fsn3.4255

**Published:** 2024-06-18

**Authors:** Cuneyt Uyak, Erdal Aglar, Burhan Ozturk, Adnan Doğan, Onur Tekin

**Affiliations:** ^1^ Department of Horticulture, Faculty of Agriculture Van Yüzüncü Yıl University Van Turkey; ^2^ Department of Horticulture, Faculty of Agriculture Ordu University Ordu Turkey

**Keywords:** antioxidant activity, phenolic compounds, total flavonoids, vitamin C

## Abstract

The present study was carried out to assess the fruit traits and phytochemical properties of 39 mulberry genotypes [*Morus alba* L. (16 white mulberry), *Morus rubra* L. (11 red mulberry), and *Morus nigra* L. (12 black mulberry)] selected in Bitlis province (Hizan) of Türkiye. Approximately, 1 kg fruit were hand‐harvested from each genotype. The fruit size, fruit color, pH, titratable acidity, soluble solids content (SSC), vitamin C, total phenolics, total flavonoids, antioxidant activity, and individual phenolic compounds were determined. The fruit weight varied between 1.80 and 5.84 g in black mulberry, 1.22 and 4.18 g in red mulberry, and 1.29 and 3.10 g in white mulberry. In white mulberry, SSC was determined to be between 19.00% and 38.86%, and in black and red mulberry it was between 11.86% and 21.90% and 12.60% and 18.90%, respectively. The titratable acidity rate, which varied depending on the species, was lower in white mulberry, but the highest acidity rate was recorded in red mulberry. Vitamin C was determined as 33.13 mg 100 g^−1^ in red mulberry, 24.10 mg 100 g^−1^ in black mulberry, and 14.03 mg 100 g^−1^ in white mulberry. Total phenolics in mulberries varied depending on the species, and the red mulberry fruit contained higher phenolic substances, followed by black and white mulberries. The total flavonoids varied between 0.27 and 7.83 g QE kg^−1^, whereas the flavonoids varied depending on the species and genotype. The highest values in terms of bioactive compounds were recorded in fruits of the 13HZN23 and 37 genotypes. Black and red mulberry fruit were found to have higher levels of individual phenolic content than the white mulberrry fruit.

## Graphical abstract

It was observed that there was a wide variation among both species and genotypes in terms of the fruit and biochemical properties examined. It was determined that the variation between genotypes in terms of biochemical properties was lower in white mulberries and higher in black and red mulberries. Genotypes 13HZH23 and 13HZN 37 showed the highest values in terms of the 12 biochemical properties examined.

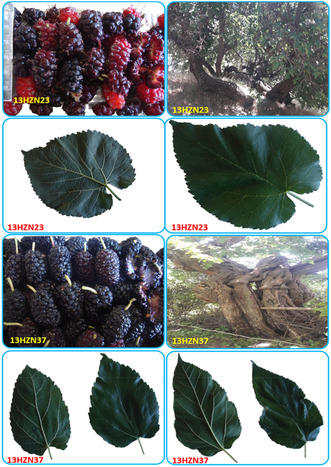

## 1 INTRODUCTION

Mulberry, which belongs to the genus *Morus* of the Moraceae family, can grow in all regions between 50° north latitude and 10° south latitude, including Asia, Europe, North and South America, and Africa, and in places up to 4000 m above sea level (Machii et al., 1999). About 100 species of the genus *Morus* have been described in the world, and 10–12 of these species are considered to grow widely (De Candolle, 1967). However, the species and varieties of mulberries produced and whose fruits are used include *Morus alba* L. (white mulberry), *Morus nigra* L. (black mulberry), and *Morus rubra* L. (red or purple mulberry) (Bellini et al., 2000).

Mulberry has the potential to attract attention in domestic and foreign markets with its use in sectors, such as food, cosmetics, health, furniture, and sericulture (Erdogan & Pirlak, 2005). In Anatolia, which is among the homelands of mulberry and has extremely suitable ecological conditions for its cultivation, mulberry species including *M. alba* L. (white mulberry), *M. nigra* L. (black mulberry), and *M. rubra* L (purple mulberry) (Ercisli & Orhan, 2007) have been cultivated and produced for more than 400 years. Türkiye (Agaoglu et al., 1995), which allows the cultivation of most fruit species due to its favorable ecological conditions, location on trade routes, and hosting many civilizations since the early ages of history, is one of the few countries in the world in terms of plant genetic resources. However, these plant genetic resources are in danger of decreasing or even disappearing due to environmental and other pressures in the regions where they are found. Their protection is important in terms of securing the crop production of the future (Karagoz et al., 2010). Improving the use of plant genetic resources for food and agriculture can be achieved by determining all the properties of the material and introducing those with superior properties into cultivation (Sehirali & Ozgen, 1987).

In this sense, the point selection studies on fruit species are of great importance. In these selection studies, the regions should be researched separately on a species basis and their characteristics should be determined. Considering that biochemical properties of fruit species vary depending on ecological factors (Serra et al., 2011), phytochemical properties as well as pomological and morphological properties should be included in selection studies. Mulberry, which is used in sectors such as food, cosmetics, health, furniture, and sericulture, is also a species rich in bioactive compounds (Munin & Edwards‐Lévy, 2011). Mulberry consumption is increasing day by day due to its positive effects on human health due to the bioactive components it contains. Although the modern white mulberry orchards have begun to be established in Turkiye in recent years, the fact that mulberry cultivation is performed in a few species and generally in the form of scattered trees offers a great genetic richness. Therefore, the phytochemical content may vary depending on the species and region. In mulberry fruit, which is a species of fruit rich in phenolic compounds, such as flavonoids, anthocyanins, and carotenoids, which have important effects on human nutrition and health, the content and concentration of these compounds vary depending on genetic and ecological factors (Munin & Edwards‐Lévy, 2011). For this reason, it is very important to determine the content of bioactive compounds in mulberry on the basis of species and region. The studies have shown that the bioactive compound content varies depending on the species (Akbulut et al., 2006; Aljane & Sdırı, 2016; Gecer et al., 2016; Kaya, 2020; Yoncaci, 2020) and black mulberry has higher values in terms of content and concentration (Gundogdu et al., 2018). However, it has been reported that the concentration of bioactive compounds varies among mulberry genotypes within the species (Gundogdu et al., 2017; Krishna et al., 2020; Yaman, 2021), but it has been suggested that geography (Khattak & Rahman, 2015) and altitude (Paunović et al., 2020) also affect the content and concentration of bioactive compounds.

There is no study on mulberry selection and determination of biochemical content in the region regarding mulberry genotypes that have found a natural distribution area in the Hizan region, which offers a great biodiversity with very different climate characteristics. Based on this idea, we aimed to determine the biochemical characteristics of *M. alba* L. (white mulberry), *M. rubra* L. (red mulberry), and *M. nigra* L. (black mulberry) species growing in the Hizan (Bitlis) region.

## 2 MATERIALS AND METHODS

### 2.1 Plant material

The research was conducted in Hizan (Bitlis, Türkiye) central district and villages where mulberry cultivation is intensive. The plant material of the study consisted of genotypes of *M. alba* L. (white mulberry), *M. rubra* L. (red mulberry), and *M. nigra* L. (black mulberry) species grown in the region. During the harvest period, approximately 1 kg fruit harvested from 39 genotypes (16 white, 11 red, and 12 black mulberry) were immediately transported in a refrigerated vehicle (15°C and 85% relative humidity (RH)) to the Horticulture Pomology laboratory of Van Yüzüncü Yıl University in plastic chalet boxes.

### 2.2 Methods

#### 2.2.1 Fruit weight, fruit sizes, and color traits

Fruit weight, the fruit harvested at 5‐day intervals for each genotype, was measured by a digital scale with a precision of 0.01 g (Radwag, Poland) and their averages were taken. Fruit width was determined by measuring with a digital caliper sensitive to 0.01 mm from the middle part of the fruit. Fruit length, the part between the stem and the tip of the fruit, was determined by measuring with a digital caliper sensitive to 0.01 mm. Fruit color was determined based on CIE L*, a*, b*, chroma (C*), and hue (*h*°) values. It was determined by taking measurements from 10 fruit using a colorimeter (Minolta, model CR–400, Tokyo, Japan) at two opposite poles of the equatorial part of the fruit.

#### 2.2.2 Soluble solids content (SSC), titratable acidity, and pH

In order to determine the titratable acidity, 20 mL of distilled water was added to 10 mL of the fruit juice sample and diluted, and the juice was titrated with 0.1 N sodium hydroxide (NaOH) in a pH meter until the pH of the fruit reached 8.1, and the titratable acidity content was calculated as percentage (%). The pH value of the fruit juice was determined with the help of a pH meter, and the SSC was determined with a digital refractometer and stated as percentage (%).

#### 2.2.3 Vitamin C, total phenolics, and total flavonoids

For the analysis of vitamin C, total flavonoids, total phenolic compounds, anthocyanin quantity, and antioxidant activity (2,2‐diphenyl‐1‐picrylhydrazyl (DPPH) and ferric ion reducing antioxidant potential (FRAP)), the mulberry samples were homogenized by being crushed with a mixer. The homogenized fruit samples were placed in falcon tubes (approximately 150–200 g) and stored at −80°C, until the specified bioactive analyses were performed. Vitamin C was determined by diluting a squeezed and filtered 5 g fruit juice sample with 50 mL oxalic acid, then dipping the ascorbic acid test kit into the diluted solution for 2 s, waiting for it to oxidize outside for 8 s, and then placing it in the test adapter of the Reflectoquant device (Merck RQflex 20) for 5 s. Total phenolics were determined by using Folin–Ciocalteu's reagent. Initially, 400 μL of fresh fruit extract was taken and 4.2 mL of distilled water was added, then 100 μL of Folin–Ciocalteu's reagent and 2% sodium bicarbonate (Na_2_CO_3_) were added and incubated for 2 h. The solution, which turned bluish after incubation, was measured in a spectrophotometer at a wavelength of 760 nm. By drawing a graph with the standard solution prepared at different concentrations of gallic acid (GA) and using the formula obtained, the absorbance results of the samples were calculated as gallic acid equivalent (GAE) g kg^−1^ fresh weight.

For total flavonoid analysis, 1 mL of appropriately diluted extract was made up to 5 mL with distilled water and 0.3 mL of 5% sodium nitrite (NaNO_2_) was added. After 5 min, 10% aluminum chloride (AlCl_3_) was added to the mixture and left for 6 min. Then, 1 M NaOH was added and the total volume was completed to 10 mL with distilled water. The absorbance of the resulting solution was measured in a spectrophotometer at 510 nm. The total flavonoids in the fruit were calculated as g quercetin equivalent (QUE) kg^−1^ fresh weight (Zhishen et al., 1999).

#### 2.2.4 Antioxidant activity (DPPH and FRAP)

2,2‐diphenyl‐1‐picrylhydrazyl (DPPH) was determined by modifying the method of Blois (1958). DPPH solution was used as free radical. The stock solutions were transferred to the test tubes to create solutions at different concentrations, respectively. The total volume of 0.5 mL of 0.1 mM ethanol solution of DPPH free radical, sample extract, and standard antioxidant solution (50–500 μg/mL) was made up to 3 mL, and the mixture was dynamically mixed and kept at room temperature for 30 min. Then, the absorbance of the mixture was measured at 517 nm. Results are given in mmol (trolox equivalent) TE kg^−1^ fresh fruit. For FRAP analysis (Ozturk et al., 2019), phosphate buffer (1.15 mL, 0.2 M, pH 6.7) was first prepared and added together with potassium ferricyanide (K_3_Fe (CN)_6_) (1.25 mL, 1%) onto 100 μL of mulberry fruit extract sample. Then, the reaction mixture was kept at 50°C for 20 min and then cooled to room temperature. Then, trichloroacetic acid [TCA, (1.25 mL, 10%)] and ferric chloride [FeCl_3_ (0.25 mL, 0.1%)] were added and mixed by vortexing for 1 min. Finally, the absorbance of the solution was read at 700 nm on an ultraviolet–visible (UV–vis) spectrophotometer. The results are given in mmol TE kg^−1^ fresh fruit.

#### 2.2.5 Total monomeric anthocyanin

Total monomeric anthocyanin (TMA) in fruit was determined using the pH difference method (Giusti et al., 1999). The extracts were prepared at pH 1.0 and 4.5 buffer and measured at wavelengths of 533 and 700 nm. Total anthocyanin amount (molar extinction coefficient of 29,600 of cyanidin 3‐glucoside) absorbances [(A520–A700) pH 1.0–(A520–A700) pH 4.5] were calculated as μg cyanidin 3 glycosides g^−1^ fw.

#### 2.2.6 Specific phenolic compounds

Homogeneously selected fresh fruit samples were precisely weighed as one gram and extracted with methyl alcohol (5 mL) in a test tube for 6 h. The resulting filtrate was analyzed by high‐pressure liquid chromatography (HPLC) (Ultimate 3000, HPLC, Thermo‐Scientific). The HPLC system was equipped with an ultraviolet (UV) detector and quaternary solvent distribution system and the readings were recorded at 280 nm. Analytes were separated with a Phenomenex Kromasil (Phenomenex, Torrance, CA, USA) 100A C18 (250 × 4.60 mm, 5 μm) column. The column temperature was maintained at 26°C using a water bath, and the mobile phase was composed of water and acetonitrile (A) containing 2.5% formic acid (B). The mobile phase flow rate was kept at 1 mL per minute and 20 μL of sample was injected, and the results of the peak areas obtained were expressed as milligrams per kilogram (mg/kg). In the research, aminobenzoic acid, p‐hydroxybenzoic acid, protocatechuic acid, (+)‐catechin, (−)‐epicatechin, rutin, caffeic acid, p‐coumaric acid, and chlorogenic acid were determined.

### 2.3 Statistical analysis

The study was conducted according to the randomized plot design with 10 replications for fruit and color characteristics and three replications for biochemical characteristics. The data obtained were subjected to variance analysis with one‐way analysis of variance (ANOVA) test using the SPSS package program. Duncan's multiple comparison test was used to determine different groups and the statistical significance level was taken as *p* < .05. Cluster analysis and principal component analysis (PCA) were performed using JMP® Pro statistical software. The “ggplot 2” package was used for principal component analysis (PCA) (Wickham, 2016). Clustering analysis was performed with the “Hierarchical Clustering‐Ward” method (Murtagh & Legendre, 2014).

## 3 RESULTS

### 3.1 Fruit weights, fruit sizes, and color traits

The significant differences were detected between species and genotypes in terms of fruit size, which is considered as fruit weight, fruit width, and fruit length. In terms of fruit weight, it was determined that black mulberry fruit were bigger, followed by red and white mulberry fruit. The highest fruit weight value in black mulberries was 5.84 g (13HZN28), and there were genotypes with 4.18 g (13HZN26) and 3.10 g (13HZN05) in red and white mulberries, respectively. In black mulberry genotypes, the fruit weight varied between 1.80 and 5.84 g, the fruit width varied between 12.27 and 18.39 mm, and the fruit length varied between 19.34 and 28.43 mm. In red mulberry genotypes, the fruit weight, fruit width, and fruit length were between 1.22 and 4.18 g, 11.26 and 15.82 mm, and 17.24 and 23.94 mm, respectively. In white mulberry genotypes, the fruit weight, fruit width, and fruit length were between 1.29 and 3.10 g, 11.52 and 15.14 mm, and 17.24 and 23.94 mm, respectively, in Table 1.

**TABLE 1:** Fruit size in mulberry genotypes in the Hizan region.

Species	Genotype	Altitude (m)	Fruit weight (g)	Fruit length (mm)	Fruit width (mm)
*Morus alba* (White mulberry)	13HZN01	1199	2.12 ± 0.23 c	22.40 ± 1.99 cd	13.61 ± 0.75 d
	13HZN02	989	1.90 ± 0.34 c	21.95 ± 1.34 d	14.05 ± 0.62 bcd
	13HZN03	985	1.82 ± 0.29 c	20.95 ± 1.19 de	13.77 ± 0.89 cd
	13HZN04	991	2.03 ± 0.33 c	21.82 ± 1.29 d	14.12 ± 0.99 bcd
	13HZN05	991	3.10 ± 0.60 a	26.46 ± 1.96 a	14.81 ± 1.02 ab
	13HZN06	1173	2.74 ± 0.46 b	24.11 ± 1.86 bc	15.14 ± 0.73 a
	13HZN07	1307	2.18 ± 0.49 c	23.96 ± 2.38 bc	13.41 ± 1.00 d
	13HZN08	1396	1.29 ± 0.23 d	18.45 ± 1.62 fg	12.23 ± 0.58 ef
	13HZN09	1390	2.70 ± 0.48 b	25.23 ± 1.88 ab	14.48 ± 0.61 abc
	13HZN10	1666	1.92 ± 0.41 c	20.68 ± 2.71 de	13.50 ± 0.74 d
	13HZN11	1571	1.29 ± 0.18 d	17.71 ± 1.30 g	11.52 ± 0.70 f
	13HZN12	1532	1.21 ± 0.22 d	17.33 ± 1.47 g	12.00 ± 0.69 ef
	13HZN13	1685	1.30 ± 0.32 d	18.96 ± 2.26 fg	12.15 ± 0.93 ef
	13HZN14	1701	1.22 ± 0.28 d	18.75 ± 1.61 fg	11.79 ± 0.73 ef
	13HZN15	1272	1.30 ± 0.30 d	18.58 ± 2.01 fg	12.01 ± 0.99 ef
	13HZN16	1350	1.92 ± 0.36 c	19.58 ± 1.84 ef	12.51 ± 0.44 e
*Morus rubra* (Red mulberry)	13HZN17	1570	3.14 ± 0.82 bcd	22.92 ± 4.36 ab	14.63 ± 1.42 abc
	13HZN18	1600	2.87 ± 0.43 cd	20.98 ± 1.95 abc	14.55 ± 1.21 abc
	13HZN19	1429	3.03 ± 0.59 cd	21.29 ± 1.60 abc	15.31 ± 1.11 abc
	13HZN20	1335	2.65 ± 0.90 d	20.14 ± 2.28 bcd	13.56 ± 2.09 c
	13HZN21	1360	3.86 ± 1.13 ab	23.73 ± 4.37 a	15.82 ± 1.57 a
	13HZN22	1262	1.22 ± 0.37 e	17.24 ± 3.33 d	11.78 ± 2.31 d
	13HZN23	1301	3.52 ± 0.88 abc	23.94 ± 6.19 a	15.60 ± 2.99 a
	13HZN24	1633	2.91 ± 1.09 cd	20.50 ± 4.13 abcd	13.74 ± 1.61 bc
	13HZN25	1620	1.68 ± 0.49 e	17.92 ± 3.26 cd	11.26 ± 1.75 d
	13HZN26	1508	4.18 ± 0.76 a	21.99 ± 2.28 ab	15.44 ± 1.16 ab
	13HZN27	1511	3.49 ± 0.87 abc	23.88 ± 1.80 a	15.10 ± 1.15 abc
*Morus nigra* (Black mulberry)	13HZN28	1225	5.84 ± 0.90 a	28.43 ± 2.80 a	18.39 ± 1.41 a
	13HZN29	991	3.56 ± 0.47 bc	23.59 ± 2.20 cde	16.52 ± 0.77 cd
	13HZN30	1481	3.58 ± 0.68 bc	20.78 ± 1.59 fg	15.99 ± 1.62 d
	13HZN31	1463	4.01 ± 0.66 b	23.10 ± 2.04 def	16.84 ± 1.91 bcd
	13HZN32	1509	5.20 ± 0.70 a	25.85 ± 2.25 bc	17.91 ± 1.35 ab
	13HZN33	1512	4.33 ± 0.70 b	25.60 ± 1.92 c	17.66 ± 0.95 abc
	13HZN34	1276	3.97 ± 0.66 b	24.87 ± 2.03 cd	16.23 ± 1.33 d
	13HZN35	1262	3.94 ± 1.03 b	25.52 ± 2.00 c	16.10 ± 1.95 d
	13HZN36	1248	1.80 ± 0.23 e	19.34 ± 1.25 g	12.27 ± 0.68 f
	13HZN37	1300	3.83 ± 1.52 b	27.88 ± 4.36 ab	14.50 ± 1.34 e
	13HZN38	1489	3.05 ± 0.63 cd	22.34 ± 2.23 ef	14.00 ± 1.14 e
	13HZN39	1458	2.63 ± 0.50 cd	21.02 ± 2.81 fg	13.51 ± 1.14 e

*Note*: Means in columns with the same letter do not differ according to Duncan's test at *p* < .05.

The fruit color was determined by L*, a*, b*, chroma (C*), and hue (*h*°) values. Fruit color values, as well as fruit size, varied depending on the species and genotype. Considering the L* value, the highest value was recorded in white mulberry, but the lowest values were obtained in black mulberry fruit. The L* value in white mulberry varied between 56.91 (13HZN10) and 66.22 (13HZN14), and there were significant differences in L* value among white mulberry genotypes. The red and black mulberry values also differed depending on the genotype, and L* values in these two species varied between 17.90 (13HZN20) and 24.26 (13HZN19) and 15.68 (13HZN37) and 18.39 (13HZN39), respectively. The highest values (10.22 to 23.52) in terms of a* value, which expresses the red color in the fruit, were naturally obtained from red mulberry genotypes. The a* value in black mulberry varied between 1.25 (13HZN31) and 5.18 (13HZN34), and in white mulberry, it varied between −2.79 (13HZN05) and −0.13 (13HZN15). There were significant differences between genotypes in terms of a* value in all three mulberry species. The b* color value, which indicates the brightness of the fruit, varied depending on the species and genotype within the species. The highest b* value was detected in white mulberry, followed by red and black mulberry. The 13HZN13 genotype had the highest value (26.79) in white mulberry, but the lowest value (17.76) was recorded in fruit of the 13HZN03 genotype. In red and black mulberry, the b* value varied between 3.60 (13HZN20) and 11.09 (13HZN19) and 1.37 (13HZN32) and 2.66 (13HZN34), respectively. The chroma (C*) and hue (*h*°) values, which vary depending on the species and genotype, were higher in white mulberry. It was determined that the chroma (C*) value was higher in red mulberry and the hue (*h*°) value was higher in black mulberry than in the other two species in Table 2.

**TABLE 2:** Fruit color treats in mulberry genotypes in the Hizan region.

Species	Genotype	L*	a*	b*	Croma (C*)	Hue (*h*°)
*Morus alba* (White mulberry)	13HZN01	62.22 ± 1.12 ef	0.79 ± 1.02 ab	19.11 ± 0.82 gh	19.46 ± 0.90 g	87.35 ± 1.14 g
	13HZN02	65.41 ± 0.87 ac	0.18 ± 1.06 ac	16.50 ± 0.65 ı	16.52 ± 0.79 h	89.73 ± 1.13 f
	13HZN03	65.32 ± 1.19 ac	−0.78 ± 0.90 af	17.76 ± 1.12 hı	17.79 ± 0.86 h	92.20 ± 1.01 d
	13HZN04	63.96 ± 0.89 cd	−0.92 ± 1.05 af	24.24 ± 1.05 cd	24.34 ± 0.97 c	94.07 ± 0.74 c
	13HZN05	60.94 ± 0.95 f	−2.79 ± 0.65 f	21.47 ± 0.84 ef	21.68 ± 0.70 df	97.24 ± 0.91 ab
	13HZN06	65.67 ± 1.10 ab	−1.04 ± 1.16 bf	21.31 ± 0.84 ef	21.35 ± 0.79 ef	92.16 ± 0.95 d
	13HZN07	64.37 ± 0.91 bc	−2.03 ± 1.59 df	25.59 ± 1.03 ac	25.77 ± 0.50 ac	95.92 ± 1.05 ab
	13HZN08	63.35 ± 0.76 de	−1.92 ± 0.74 df	26.11 ± 1.08 ab	26.23 ± 0.98 ab	94.06 ± 0.66 c
	13HZN09	61.83 ± 0.52 ef	−2.72 ± 0.87 ef	25.31 ± 0.91 ac	25.52 ± 0.65 bc	95.79 ± 1.06 b
	13HZN10	56.91 ± 1.21 h	−0.72 ± 1.14 ae	24.45 ± 0.87 bc	24.52 ± 1.09 c	91.84 ± 0.96 de
	13HZN11	62.22 ± 0.67 ef	0.79 ± 1.04 ab	19.03 ± 0.79 gh	20.47 ± 0.48 fg	87.35 ± 0.85 g
	13HZN12	63.12 ± 0.57 de	−0.82 ± 1.07 af	25.12 ± 0.92 ac	25.21 ± 0.73 bc	91.69 ± 0.84 de
	13HZN13	66.03 ± 0.47 a	−2.30 ± 0.71 ef	26.79 ± 0.84 a	27.09 ± 0.91 a	97.53 ± 1.11 a
	13HZN14	66.22 ± 1.17 a	−1.26 ± 1.20 cf	22.18 ± 0.99 e	22.37 ± 0.80 de	92.65 ± 0.79 cd
	13HZN15	60.88 ± 0.67 f	−0.13 ± 0.53 ad	22.86 ± 0.89 de	22.88 ± 0.94 d	90.26 ± 0.73 ef
	13HZN16	58.53 ± 0.57 g	1.03 ± 1.13 a	20.35 ± 0.72 fg	20.45 ± 0.55 fg	86.85 ± 0.93 g
*Morus rubra* (Red mulberry)	13HZN17	18.78 ± 0.87 d	12.35 ± 1.31 e	4.45 ± 0.87 e	13.15 ± 1.02 f	20.80 ± 1.01 ce
	13HZN18	22.48 ± 0.90 c	20.04 ± 0.90 b	9.04 ± 1.08 b	22.06 ± 0.68 b	23.05 ± 1.07 ab
	13HZN19	24.26 ± 0.88 b	23.52 ± 0.50 a	11.09 ± 0.94 a	26.15 ± 0.53 a	23.98 ± 1.02 a
	13HZN20	17.90 ± 0.66 d	10.77 ± 0.61 ef	3.60 ± 0.80 e	11.36 ± 1.03 fg	18.49 ± 1.16 f
	13HZN21	22.31 ± 1.04 c	14.37 ± 0.94 d	5.91 ± 0.69 d	15.55 ± 1.02 e	21.96 ± 1.00 bd
	13HZN22	27.78 ± 1.11 a	18.65 ± 0.77 b	7.59 ± 0.60 c	20.16 ± 0.98 c	22.30 ± 1.12 ac
	13HZN23	18.07 ± 1.10 d	10.75 ± 1.07 ef	3.78 ± 0.53 e	11.62 ± 1.19 fg	19.50 ± 1.20 ef
	13HZN24	19.15 ± 1.12 d	11.86 ± 0.98 e	4.24 ± 0.84 e	12.65 ± 1.20 fg	22.18 ± 1.17 ac
	13HZN25	19.62 ± 1.03 d	16.07 ± 0.82 c	6.61 ± 1.03 cd	17.42 ± 0.57 d	20.81 ± 0.89 ce
	13HZN26	17.98 ± 0.53 d	11.41 ± 0.609 ef	3.94 ± 0.48 e	12.09 ± 0.94 fg	20.10 ± 0.72 df
	13HZN27	18.91 ± 0.95 d	10.22 ± 0.688 f	3.79 ± 0.83 e	11.19 ± 1.09 g	19.17 ± 1.04 ef
*Morus nigra* (Black mulberry)	13HZN28	16.59 ± 1.48 ac	1.35 ± 0.59 d	1.52 ± 0.60 a	2.05 ± 0.82 c	47.65 ± 1.12 b
	13HZN29	17.06 ± 1.13 ac	3.85 ± 1.19 b	2.34 ± 0.60 a	4.55 ± 1.23 b	33.70 ± 1.11 d
	13HZN30	17.29 ± 0.96 ac	1.70 ± 0.70 cd	1.51 ± 0.55 a	2.34 ± 1.11 c	46.71 ± 1.31 b
	13HZN31	16.81 ± 1.08 ac	1.25 ± 0.60 d	1.43 ± 0.40 a	1.94 ± 0.45 c	50.31 ± 1.20 a
	13HZN32	16.81 ± 1.02 ac	1.53 ± 0.75 d	1.37 ± 0.83 a	2.09 ± 0.87 c	42.75 ± 1.16 c
	13HZN33	16.43 ± 0.89 bc	2.89 ± 0.77 bd	1.58 ± 0.63 a	3.37 ± 0.78 bc	34.80 ± 1.02 d
	13HZN34	17.80 ± 0.94 ab	5.18 ± 1.32 a	2.66 ± 1.52 a	6.84 ± 1.62 a	25.03 ± 1.54 g
	13HZN35	16.13 ± 1.00 bc	3.87 ± 1.40 b	2.12 ± 1.02 a	4.46 ± 0.81 b	32.92 ± 1.05 d
	13HZN36	16.06 ± 0.80 bc	1.57 ± 0.70 d	1.38 ± 0.46 a	2.11 ± 0.87 c	43.57 ± 0.62 c
	13HZN37	15.68 ± 1.10 c	3.33 ± 0.98 bc	1.91 ± 0.68 a	3.90 ± 1.18 bc	34.77 ± 1.48 d
	13HZN38	17.57 ± 0.66 ac	4.46 ± 1.18 b	1.80 ± 0.85 a	4.87 ± 1.05 b	27.86 ± 1.09 f
	13HZN39	18.39 ± 0.61 a	3.97 ± 0.81 b	2.02 ± 0.94 a	4.57 ± 1.38 b	30.76 ± 1.50 e

*Note*: Means in columns with the same letter do not differ according to Duncan's test at *p* < .05.

### 3.2 Soluble solids content (SSC), titratable acidity, pH, and vitamin C

The SSC in white mulberry varied between 19.00% and 38.86%, and it was between 11.86% and 21.90% and 12.60% and 18.90% in black and red mulberry, respectively. The amount of SSC varied significantly among species and intraspecific genotypes in Table 3. The titratable acidity, which varies depending on the species, was lower in white mulberry with a high SSC rate, but the highest acidity rate was detected in red mulberry. There were significant differences in acidity ratio among genotypes within species. The titratable acidity in white mulberry varied between 0.16% (13HZN06) and 0.30 (13HZN12), in black and red mulberry it varied between 0.20% (13HZN31) and 0.56 (13HZN35) and 0.18% (13HZN22) and 2.39 (13HZN19), respectively (Table 3). The pH in fruit varied depending on the species and genotype within the species. The highest pH value was detected in white mulberry, followed by black and red mulberry. The 13HZN10 genotype had the highest value (6.66) in white mulberry, but the lowest value (6.09) was recorded in fruit of the 13HZN01 genotype. In red and black mulberry, pH values varied between 3.75 (13HZN25) and 5.54 (13HZN26 and 27) and 4.59 (13HZN29) and 6.13 (13HZN28), respectively (Table 3). In red and black mulberry, pH values varied between 3.75 (13HZN25) and 5.54 (13HZN26 and 27) and 4.59 (13HZN29) and 6.13 (13HZN28), respectively (Table 3). There were significant differences in the vitamin C content of the fruit depending on the species. There was a genotype with 33.13 mg 100 g^−1^ vitamin C content in red mulberry, but the highest vitamin C content was recorded as 24.10 mg 100 g^−1^ in black mulberry and 14.03 mg 100 g^−1^ in white mulberry. There were also significant differences in vitamin C content between genotypes within the species. Vitamin C content of the genotypes was determined to be between 5.20 and 14.03 mg 100 g^−1^ in white mulberry, 6.50 and 33.13 mg 100 g^−1^ in red mulberry, and 13.13 and 24.10 mg 100 g^−1^ in black mulberry. There was a greater difference between genotypes in red mulberry in Table 3.

**TABLE 3:** Soluble solids content (SSC), titratable acidity, pH, and vitamin C in mulberry genotypes in the Hizan region.

Species	Genotype	SSC (%)	Titratable acidity (%)	pH	Vitamin C (mg 100 g^−1^)
*Morus alba* (White mulberry)	13HZN01	28.93 ± 0.05 e	0.17 ± 0.00 gh	6.09 ± 0.01 j	7.06 ± 0.15 h
	13HZN02	38.86 ± 0.11 a	0.27 ± 0.00 b	6.11 ± 0.01 i	9.40 ± 0.20 f
	13HZN03	29.03 ± 0.20 e	0.20 ± 0.00 def	6.23 ± 0.00 h	7.03 ± 0.05 h
	13HZN04	27.33 ± 0.20 f	0.26 ± 0.00 b	6.20 ± 0.00 ı	11.23 ± 0.35 d
	13HZN05	23.53 ± 0.05 k	0.23 ± 0.01 c	6.37 ± 0.00 g	5.20 ± 0.26 i
	13HZN06	24.93 ± 0.20 i	0.16 ± 0.00 h	6.52 ± 0.00 d	6.30 ± 0.10 ı
	13HZN07	29.36 ± 0.11 d	0.26 ± 0.00 b	6.36 ± 0.00 g	10.10 ± 0.30 e
	13HZN08	30.33 ± 0.05 c	0.23 ± 0.01 c	6.57 ± 0.01 c	7.43 ± 0.05 h
	13HZN09	25.83 ± 0.05 h	0.21 ± 0.00 cd	6.65 ± 0.00 a	13.26 ± 0.25 b
	13HZN10	24.30 ± 0.10 j	0.17 ± 0.02 gh	6.66 ± 0.01 a	8.83 ± 0.15 g
	13HZN11	25.53 ± 0.05 ı	0.21 ± 0.00 cde	6.63 ± 0.00 b	11.13 ± 0.35 d
	13HZN12	31.93 ± 0.11 b	0.30 ± 0.02 a	6.48 ± 0.00 e	12.13 ± 0.45 c
	13HZN13	26.43 ± 0.05 g	0.26 ± 0.00 b	6.57 ± 0.00 c	14.03 ± 0.25 a
	13HZN14	25.33 ± 0.15 ı	0.19 ± 0.00 efg	6.48 ± 0.01 e	10.13 ± 0.35 e
	13HZN15	21.16 ± 0.28 l	0.18 ± 0.00 fgh	6.41 ± 0.00 f	13.56 ± 0.45 b
	13HZN16	19.00 ± 0.00 m	0.19 ± 0.03 efg	6.57 ± 0.02 c	9.26 ± 0.15 fg
*Morus rubra* (Red mulberry)	13HZN17	15.60 ± 0.72 d	1.40 ± 0.01 e	4.29 ± 0.01 d	17.30 ± 0.40 d
	13HZN18	15.03 ± 0.57 e	2.07 ± 0.01 c	3.94 ± 0.00 f	14.93 ± 0.15 f
	13HZN19	11.30 ± 0.10 h	2.39 ± 0.03 a	3.78 ± 0.00 g	18.73 ± 0.25 c
	13HZN20	18.90 ± 0.26 a	1.71 ± 0.00 d	4.25 ± 0.01 d	13.86 ± 0.25 g
	13HZN21	15.96 ± 0.15 d	2.14 ± 0.00 b	4.04 ± 0.00 e	17.46 ± 0.45 d
	13HZN22	13.10 ± 0.00 f	0.18 ± 0.00 ı	4.64 ± 0.00 b	16.63 ± 0.30 e
	13HZN23	18.36 ± 0.05 b	2.17 ± 0.07 b	4.07 ± 0.01 e	33.13 ± 0.45 a
	13HZN24	16.56 ± 0.05 c	0.93 ± 0.00 g	4.45 ± 0.01 c	24.63 ± 0.25 b
	13HZN25	12.60 ± 0.10 g	1.02 ± 0.00 f	3.75 ± 0.01 g	11.76 ± 0.05 h
	13HZN26	16.03 ± 0.11 d	0.39 ± 0.00 h	5.54 ± 0.06 a	13.53 ± 0.35 g
	13HZN27	16.03 ± 0.11 d	0.39 ± 0.00 h	5.54 ± 0.06 a	6.50 ± 0.10 ı
*Morus nigra* (Black mulberry)	13HZN28	18.23 ± 0.05 e	0.23 ± 0.00 i	6.13 ± 0.02 b	17.73 ± 0.90 bcd
	13HZN29	21.90 ± 0.00 a	1.21 ± 0.00 a	4.59 ± 0.00 j	21.13 ± 0.35 abc
	13HZN30	19.83 ± 0.05 c	0.28 ± 0.01 h	5.58 ± 0.00 d	21.43 ± 0.35 abc
	13HZN31	20.60 ± 0.10 b	0.20 ± 0.01 j	6.24 ± 0.01 a	13.13 ± 0.65 d
	13HZN32	13.33 ± 0.20 ı	0.25 ± 0.00 ı	5.92 ± 0.00 c	21.13 ± 0.35 abc
	13HZN33	14.13 ± 0.05 h	0.37 ± 0.00 f	5.20 ± 0.01 h	13.96 ± 0.05 d
	13HZN34	12.20 ± 0.10 i	0.52 ± 0.00 c	4.99 ± 0.01 ı	16.13 ± 0.35 cd
	13HZN35	11.86 ± 0.28 j	0.56 ± 0.00 b	4.82 ± 0.00 i	21.30 ± 0.20 abc
	13HZN36	14.80 ± 0.10 g	0.37 ± 0.00 f	5.25 ± 0.05 g	14.53 ± 0.15 d
	13HZN37	14.36 ± 0.40 h	0.47 ± 0.00 d	5.39 ± 0.02 f	22.36 ± 0.35 ab
	13HZN38	18.86 ± 0.05 d	0.33 ± 0.00 g	5.50 ± 0.00 e	24.10 ± 0.10 a
	13HZN39	15.76 ± 0.40 f	0.41 ± 0.00 e	5.17 ± 0.00 h	17.03 ± 0.45 bcd

*Note*: Means in columns with the same letter do not differ according to Duncan's test at *p* < .05.

### 3.3 Total phenolics, total flavonoids, total anthocyanin, and antioxidant activity

Total phenolic content in mulberry varied depending on the species. The red mulberry fruit contained higher phenolic substances, which is followed by black and white mulberry. It was also determined that there were differences in phenolic substance content between genotypes within the species. Total phenolic content in white mulberry genotypes was recorded as 0.44 (13HZN07) to 3.35 g GAE kg^−1^ (13HZN13), in red and black mulberry genotypes it was 1.51 (13HZN22) to 11.32 g GAE kg^−1^ (13HZN23) and 0.95 (13HZN33) to 10.99 g GAE kg^−1^, respectively. It was seen that there were huge differences between genotypes in all three species in Table 4. The lowest amount of total flavonoids in the genotypes was determined as 0.27 g QE kg^−1^ (13HZN05), but the highest amount was 7.83 g QE kg^−1^ (13HZN23). The amount of flavonoids varied depending on the species and genotype within the species. Red mulberry genotypes have higher amounts of flavonoids. The significant differences occurred between genotypes within this species, and the amount of flavonoids varied between 1.10 and 7.83 g QE kg^−1^. The amount of flavonoids in white mulberry varied between 0.27 and 1.16 g QE kg^−1^, and in black mulberry it was found to be between 0.70 and 6.15 g QE kg^−1^ in Table 4. The significant differences occurred in terms of antioxidant activity determined through DPPH and FRAP values, depending on the species and genotypes within the species. The amount of flavonoids in white mulberry varied between 0.27 and 1.16 g QE kg^−1^, and in black mulberry it was found to be between 0.70 and 6.15 in Table 4. The significant differences occurred in terms of antioxidant activity determined through DPPH and FRAP values, depending on the species and genotypes within the species. The highest and lowest values were found to be at similar levels in red and black mulberry, but there were significant differences between genotypes in both species. In general, the highest values for all bioactive compounds were recorded in fruits belonging to 13HZN 23 and 37 genotypes (Table 4). In this sense, we believe that these genotypes should be taken into consideration.

**TABLE 4:** Total phenolics, total flavonoid, total anthocyanin, and antioxidant activity in mulberry genotypes in the Hizan region.

Species	Genotype	Total phenolic^a^	Total flavonoid^b^	DPPH^c^	FRAP^c^	Total anthocyanin^d^
*Morus alba* (White mulberry)	13HZN01	1.40 ± 0.04 ef	0.29 ± 0.05 hı	0.33 ± 0.03 i	26.57 ± 0.87 f	0.00
	13HZN02	1.70 ± 0.12 d	0.48 ± 0.10 f	0.57 ± 0.03 ghı	36.29 ± 0.50 b	0.00
	13HZN03	1.16 ± 0.07 g	0.80 ± 0.06 c	1.14 ± 0.09 c	42.27 ± 0.84 a	0.00
	13HZN04	0.68 ± 0.06 ı	0.39 ± 0.02 fgh	0.67 ± 0.08 efg	27.45 ± 1.96 ef	0.00
	13HZN05	1.30 ± 0.08 f	0.27 ± 0.02 ı	0.28 ± 0.03 i	33.28 ± 1.88 c	0.00
	13HZN06	0.68 ± 0.04 ı	0.47 ± 0.08 f	0.52 ± 0.02 ı	14.35 ± 1.79 i	0.00
	13HZN07	0.44 ± 0.02 i	0.37 ± 0.03 gh	0.54 ± 0.05 hı	15.82 ± 1.10 ıi	0.00
	13HZN08	0.68 ± 0.03 ı	0.58 ± 0.05 e	0.91 ± 0.04 d	13.98 ± 0.52 i	0.00
	13HZN09	1.17 ± 0.11 g	0.96 ± 0.06 b	1.31 ± 0.11 b	23.63 ± 3.00 g	0.00
	13HZN10	0.92 ± 0.03 h	0.68 ± 0.05 d	1.27 ± 0.02 b	16.63 ± 0.21 hı	0.00
	13HZN11	1.45 ± 0.06 e	1.16 ± 0.07 a	1.50 ± 0.09 a	31.26 ± 1.28 cd	0.00
	13HZN12	2.01 ± 0.09 c	0.61 ± 0.03 de	0.70 ± 0.03 ef	29.44 ± 0.58 de	0.00
	13HZN13	3.35 ± 0.01 a	0.42 ± 0.02 fg	0.99 ± 0.05 d	29.83 ± 0.05 d	0.00
	13HZN14	2.18 ± 0.12 b	0.38 ± 0.00 fgh	0.77 ± 0.06 e	29.26 ± 0.85 de	0.00
	13HZN15	0.95 ± 0.02 h	0.67 ± 0.02 de	0.65 ± 0.05 fgh	24.37 ± 0.27 g	0.00
	13HZN16	0.70 ± 0.07 ı	0.31 ± 0.03 hı	0.53 ± 0.03 hı	18.43 ± 0.95 h	0.00
*Morus rubra* (Red mulberry)	13HZN17	2.62 ± 0.21 f	1.70 ± 0.20 g	2.08 ± 0.08 e	4.40 ± 0.04 g	0.61 ± 0.20 g
	13HZN18	4.04 ± 0.19 e	2.58 ± 0.06 e	3.26 ± 0.13 d	6.33 ± 0.06 e	1.37 ± 0.37 f
	13HZN19	4.36 ± 0.17 de	2.25 ± 0.06 f	1.36 ± 0.13 f	4.13 ± 0.03 h	0.50 ± 0.24 g
	13HZN20	4.94 ± 0.17 cd	2.86 ± 0.08 d	2.16 ± 0.03 e	4.52 ± 0.04 g	1.62 ± 0.23 f
	13HZN21	4.76 ± 0.06 d	3.63 ± 0.18 c	2.14 ± 0.07 e	6.22 ± 0.08 e	2.84 ± 0.38 e
	13HZN22	1.51 ± 0.09 g	1.10 ± 0.10 h	1.15 ± 0.08 f	2.47 ± 0.11 ı	1.61 ± 0.22 f
	13HZN23	11.32 ± 1.10 a	7.83 ± 0.12 a	5.68 ± 0.01 a	25.36 ± 0.25 a	10.19 ± 0.37 a
	13HZN24	7.86 ± 0.15 b	4.08 ± 0.09 b	5.56 ± 0.06 a	13.22 ± 0.21 b	7.84 ± 0.42 b
	13HZN25	5.54 ± 0.05 c	2.72 ± 0.99 de	4.87 ± 0.27 b	9.74 ± 0.29 c	5.69 ± 0.52 c
	13HZN26	7.40 ± 0.04 b	3.43 ± 0.10 c	3.76 ± 0.10 c	8.48 ± 0.18 d	3.76 ± 0.54 d
	13HZN27	3.01 ± 0.10 f	2.31 ± 0.22 f	3.45 ± 0.20 d	5.63 ± 0.90 f	3.65 ± 0.21 d
*Morus nigra* (Black mulberry)	13HZN28	3.60 ± 0.10 cd	3.16 ± 0.05 c	4.00 ± 0.00 c	7.00 ± 0.00 e	3.41 ± 0.17 e
	13HZN29	3.53 ± 0.11 cd	1.70 ± 0.08 f	3.26 ± 0.11 e	4.79 ± 0.05 g	1.66 ± 0.17 g
	13HZN30	4.47 ± 0.03 c	2.49 ± 0.07 d	3.59 ± 0.10 d	5.07 ± 0.07 fg	1.55 ± 0.08 g
	13HZN31	6.69 ± 0.05 b	3.04 ± 0.04 c	5.45 ± 0.05 b	8.71 ± 0.20 d	8.20 ± 0.34 b
	13HZN32	6.65 ± 1.58 b	3.76 ± 0.20 b	5.69 ± 0.10 a	11.78 ± 0.43 c	5.57 ± 0.14 d
	13HZN33	0.95 ± 0.01 e	0.70 ± 0.18 h	1.50 ± 0.14 f	2.21 ± 0.19 h	0.47 ± 0.04 h
	13HZN34	2.57 ± 0.42 d	1.33 ± 0.15 g	1.01 ± 0.02 g	2.70 ± 0.05 h	0.54 ± 0.03 h
	13HZN35	3.80 ± 1.35 cd	2.22 ± 0.09 de	3.08 ± 0.09 e	5.17 ± 0.17 fg	0.51 ± 0.03 h
	13HZN36	5.72 ± 0.22 b	1.93 ± 0.08 ef	5.71 ± 0.31 a	8.81 ± 0.10 d	7.70 ± 0.24 c
	13HZN37	10.99 ± 1.04 a	6.15 ± 0.40 a	5.57 ± 0.11 ab	20.85 ± 0.96 b	11.59 ± 0.41 a
	13HZN38	3.85 ± 0.16 cd	1.73 ± 0.29 f	3.60 ± 0.09 d	6.67 ± 0.14 ef	2.08 ± 0.18 f
	13HZN39	3.26 ± 0.37 cd	1.95 ± 0.55 ef	1.06 ± 0.06 g	24.92 ± 3.04 a	0.53 ± 0.02 h

*Note*: Means in columns with the same letter do not differ according to Duncan's test at *p* < .05.

^**a**^ g GAE kg^−1^ FW.

^**b**^ g QE kg^−1^ FW.

^**c**^ mmol TE kg^−1^ FW.

^**d**^ g cyanidin 3‐glucoside kg^−1^FW.

### 3.4 Specific phenolic compounds

In the present study, the phenolic compounds, such as aminobenzoic acid, protocatechuic acid, epicatechin, p‐coumaric acid, rutin, caffeic acid, p‐hydroxybenzoic acid, chlorogenic acid, and catechin, were detected. The significant differences were detected in individual phenolic content in fruit according to the species and type of compound. Some bioactive compounds were not found in significant amounts in some genotypes. However, there were significant differences in rates between genotypes. The white mulberry does not have appreciable caffeic and chlorogenic acid content, but when considering the highest content across genotypes in other individual phenolics, p‐hydroxybenzoic acid was the phenolic compound with the highest concentration (3.82 mg kg^−1^) and it was followed by rutin (2.03 mg kg^−1^), catechin (1.56 mg kg^−1^), aminobenzoic acid (1.39 mg kg^−1^), protocatechuic acid (0.41 mg kg^−1^), p‐coumaric acid (0.25 mg kg^−1^), and epicatechin (0.16 mg kg^−1^), respectively. Hydroxybenzoic acid could not be detected in red mulberry. However, very high amounts of epicatechin and rutin content have been recorded in this species. The epicatechin content was recorded as 99.74 mg kg^−1^ in the 13HZN23 genotype of red mulberry, whereas the rutin amount varied between 9.34 (13HZN19) and 38.51 mg kg^−1^ (13HZN23) in the genotypes in Table 5. The very significant amounts of p‐hydroxybenzoic acid (140.11 mg kg^−1^), caffeic acid (69.68 mg kg^−1^), and rutin (50.67 mg kg^−1^) were detected in the fruits of black mulberry genotypes, and there were no differences between the genotypes in terms of these three compounds. In terms of quantity, after these three compounds in black mulberry, the order was protocatechuic acid (3.95 mg kg^−1^), chlorogenic acid (3.80 mg kg^−1^), aminobenzoic acid (1.29 mg kg^−1^), p‐coumaric acid (0.89 mg kg^−1^), and epicatechin (0.72 mg kg^−1^). Catechin could not be detected in this species. It was seen that black and red mulberry fruit had higher levels of individual phenolic content than white mulberry fruit (Table 5). Catechin could not be detected in this species. When the data were evaluated, it was seen that black and red mulberry fruit had higher levels of individual phenolic content than white mulberry fruit in Table 5.

**TABLE 5:** Specific phenolic componds content in mulberry genotypes in the Hizan region (mg kg^−1^).

Species	Genotype	Amino‐benzoic acid	Protocatechuik acid	Epicatechin	p‐coumaric acid	Rutin
*Morus alba* (White mulberry)	13HZN01	0.20 ± 0.02 f	0.07 ± 0.03 f	0.01 ± 0.00 c	0.00	0.23 ± 0.10 d
	13HZN02	0.27 ± 0.07 f	0.08 ± 0.01 ef	0.01 ± 0.00 c	0.17 ± 0.03 b	0.17 ± 0.07 de
	13HZN03	0.18 ± 0.07 f	0.06 ± 0.04 f	0.00	0.02 ± 0.00 de	0.00
	13HZN04	0.87 ± 0.04 c	0.00	0.02 ± 0.01 c	0.00	0.00
	13HZN05	0.59 ± 0.09 d	0.16 ± 0.04 bc	0.16 ± 0.04 a	0.00	0.00
	13HZN06	1.39 ± 0.04 a	0.16 ± 0.03 bc	0.00	0.00	0.00
	13HZN07	0.57 ± 0.02 d	0.06 ± 0.02 f	0.03 ± 0.02 c	0.00	0.00
	13HZN08	0.55 ± 0.06 d	0.09 ± 0.01 def	0.03 ± 0.00 c	0.00	0.12 ± 0.02 e
	13HZN09	0.66 ± 0.06 d	0.18 ± 0.05 bc	0.00	0.00	0.95 ± 0.05 b
	13HZN10	0.59 ± 0.05 d	0.13 ± 0.03 cde	0.12 ± 0.07 b	0.25 ± 0.05 a	0.13 ± 0.03 e
	13HZN11	0.60 ± 0.07 d	0.19 ± 0.02 b	0.00	0.14 ± 0.03 b	0.32 ± 0.04 c
	13HZN12	0.56 ± 0.08 d	0.14 ± 0.01 bcd	0.00	0.08 ± 0.01 c	0.31 ± 0.04 c
	13HZN13	1.14 ± 0.09 b	0.41 ± 0.07 a	0.00	0.16 ± 0.06 b	2.03 ± 0.10 a
	13HZN14	0.41 ± 0.03 e	0.08 ± 0.03 ef	0.02 ± 0.01 c	0.04 ± 0.03 cde	0.00
	13HZN15	0.28 ± 0.03 f	0.00	0.02 ± 0.01 c	0.05 ± 0.02 cd	0.00
	13HZN16	0.20 ± 0.04 f	0.00	0.00	0.05 ± 0.01 cd	0.00
*Morus rubra* (Red mulberry)	13HZN17	0.30 ± 0.06 ef	0.80 ± 0.04 f	0.61 ± 0.08 i	1.03 ± 0.05 a	14.31 ± 0.06 f
	13HZN18	0.36 ± 0.06 e	1.05 ± 0.08 d	4.52 ± 0.05 f	0.65 ± 0.08 b	20.50 ± 0.05 b
	13HZN19	0.21 ± 0.03 f	0.34 ± 0.04 ı	3.59 ± 0.10 g	0.23 ± 0.04 de	9.34 ± 0.03 ı
	13HZN20	0.79 ± 0.08 b	1.38 ± 0.08 c	18.16 ± 0.08 e	0.47 ± 0.07 c	18.72 ± 0.10 c
	13HZN21	0.66 ± 0.08 c	0.94 ± 0.07 e	24.57 ± 0.06 c	0.30 ± 0.05 d	10.40 ± 0.10 h
	13HZN22	0.40 ± 0.01 de	0.59 ± 0.03 g	1.10 ± 0.05 h	0.16 ± 0.01 ef	14.26 ± 0.09 f
	13HZN23	0.97 ± 0.07 a	2.02 ± 0.05 b	99.74 ± 0.10 a	0.68 ± 0.10 b	38.51 ± 0.04 a
	13HZN24	0.31 ± 0.06 ef	0.94 ± 0.01 e	30.00 ± 0.08 b	0.32 ± 0.08 d	17.14 ± 0.10 d
	13HZN25	0.11 ± 0.03 g	0.49 ± 0.02 h	24.37 ± 0.10 d	0.09 ± 0.01 f	9.39 ± 0.09 ı
	13HZN26	0.38 ± 0.05 e	0.84 ± 0.06 f	18.19 ± 0.09 e	0.29 ± 0.09 d	13.74 ± 0.07 g
	13HZN27	0.49 ± 0.07 d	2.51 ± 0.07 a	0.84 ± 0.06 ı	0.16 ± 0.04 ef	15.73 ± 0.10 e
*Morus nigra* (Black mulberry)	13HZN28	0.51 ± 0.02 b	2.26 ± 0.03 b	0.45 ± 0.10 b	0.00	19.69 ± 0.09 b
	13HZN29	0.00	0.00	0.00	0.00	0.00
	13HZN30	0.36 ± 0.06 c	1.26 ± 0.04 de	0.23 ± 0.03 cd	0.00	6.47 ± 0.03 e
	13HZN31	0.37 ± 0.05 c	1.53 ± 0.03 c	0.29 ± 0.08 c	0.03 ± 0.02 b	15.12 ± 0.06 c
	13HZN32	0.36 ± 0.02 c	1.30 ± 0.08 d	0.28 ± 0.06 c	0.00	0.00
	13HZN33	0.32 ± 0.03 c	1.20 ± 0.10 de	0.00	0.00	0.00
	13HZN34	0.23 ± 0.01 d	1.19 ± 0.09 e	0.31 ± 0.05 c	0.04 ± 0.03 b	6.75 ± 0.05 d
	13HZN35	0.14 ± 0.04 e	0.77 ± 0.05 f	0.16 ± 0.02 de	0.00	5.29 ± 0.10 f
	13HZN36	0.07 ± 0.01 f	0.51 ± 0.04 g	0.02 ± 0.00 f	0.00	1.76 ± 0.02 h
	13HZN37	1.29 ± 0.05 a	3.95 ± 0.05 a	0.72 ± 0.10 a	0.89 ± 0.09 a	50.67 ± 0.07 a
	13HZN38	0.09 ± 0.03 ef	0.34 ± 0.06 h	0.08 ± 0.01 ef	0.00	2.16 ± 0.02 g
	13HZN39	0.00	0.00	0.00	0.00	0.00

Species	Genotype	Caffeic acid	p‐Hydroxybenzoic acid	Chlorogenic acid	(+)‐Catechin
*Morus alba* (White mulberry)	13HZN01	0.00	0.47 ± 0.05 h	0.00	0.00
	13HZN02	0.00	0.36 ± 0.04 ı	0.00	0.11 ± 0.05 fg
	13HZN03	0.00	0.36 ± 0.03 ı	0.00	0.07 ± 0.02 gh
	13HZN04	0.00	0.43 ± 0.07 hı	0.00	0.08 ± 0.03 gh
	13HZN05	0.00	0.64 ± 0.10 fg	0.00	0.11 ± 0.04 fg
	13HZN06	0.00	0.71 ± 0.09 ef	0.00	0.18 ± 0.05 f
	13HZN07	0.00	0.57 ± 0.07 g	0.00	0.17 ± 0.05 f
	13HZN08	0.00	0.72 ± 0.02 ef	0.00	0.27 ± 0.07 e
	13HZN09	0.00	1.94 ± 0.04 b	0.00	0.31 ± 0.03 e
	13HZN10	0.00	0.64 ± 0.10 fg	0.00	0.49 ± 0.05 c
	13HZN11	0.00	1.38 ± 0.02 c	0.00	0.78 ± 0.08 b
	13HZN12	0.00	0.99 ± 0.09 d	0.00	0.41 ± 0.06 d
	13HZN13	0.00	3.82 ± 0.03 a	0.00	1.56 ± 0.06 a
	13HZN14	0.00	0.78 ± 0.04 e	0.00	0.33 ± 0.03 e
	13HZN15	0.00	0.25 ± 0.03 i	0.00	0.00
	13HZN16	0.00	0.21 ± 0.01 i	0.00	0.00
*Morus rubra* (Red mulberry)	13HZN17	9.33 ± 0.09 h	0.00	42.09 ± 0.06 d	0.08 ± 0.02 f
	13HZN18	33.86 ± 0.06 e	0.00	50.72 ± 0.05 b	0.24 ± 0.04 d
	13HZN19	16.74 ± 0.10 g	0.00	57.97 ± 0.07 a	0.20 ± 0.05 de
	13HZN20	35.36 ± 0.10 d	0.00	46.26 ± 0.06 c	0.32 ± 0.02 c
	13HZN21	38.95 ± 0.03 c	0.00	18.75 ± 0.03 h	0.20 ± 0.05 de
	13HZN22	0.25 ± 0.03 i	0.00	14.95 ± 0.10 ı	0.79 ± 0.09 a
	13HZN23	40.31 ± 0.03 b	0.00	41.57 ± 0.07 e	0.16 ± 0.06 def
	13HZN24	44.39 ± 0.09 a	0.00	13.07 ± 0.07 i	0.15 ± 0.05 ef
	13HZN25	31.89 ± 0.04 f	0.00	27.60 ± 0.10 f	0.00
	13HZN26	33.96 ± 0.06 e	0.00	19.90 ± 0.08 g	0.09 ± 0.02 f
	13HZN27	1.31 ± 0.10 ı	0.00	0.81 ± 0.03 j	0.50 ± 0.05 b
*Morus nigra* (Black mulberry)	13HZN28	16.93 ± 0.03 c	108.54 ± 0.34 b	0.84 ± 0.45 b	0.00
	13HZN29	0.01 ± 0.00 ı	0.00	0.00	0.00
	13HZN30	1.24 ± 0.10 f	26.94 ± 0.10 g	0.00	0.00
	13HZN31	18.59 ± 0.03 b	45.14 ± 0.04 f	0.00	0.00
	13HZN32	0.31 ± 0.05 h	49.50 ± 0.07 e	0.00	0.00
	13HZN33	0.00	78.94 ± 0.04 c	3.80 ± 0.08 a	0.00
	13HZN34	2.12 ± 0.10 e	55.87 ± 0.09 d	0.00	0.00
	13HZN35	5.22 ± 0.02 d	45.13 ± 0.03 f	0.00	0.00
	13HZN36	0.58 ± 0.07 g	16.87 ± 0.07 ı	0.00	0.00
	13HZN37	69.68 ± 0.08 a	140.11 ± 0.10 a	0.41 ± 0.04 c	0.00
	13HZN38	0.00	20.45 ± 0.06 h	0.00	0.00
	13HZN39	0.00	0.00	0.00	0.00

*Note*: Means in columns with the same letter do not differ according to Duncan's test at *p* < .05.

### 3.5 Principal component analysis

**FIGURE 1:**
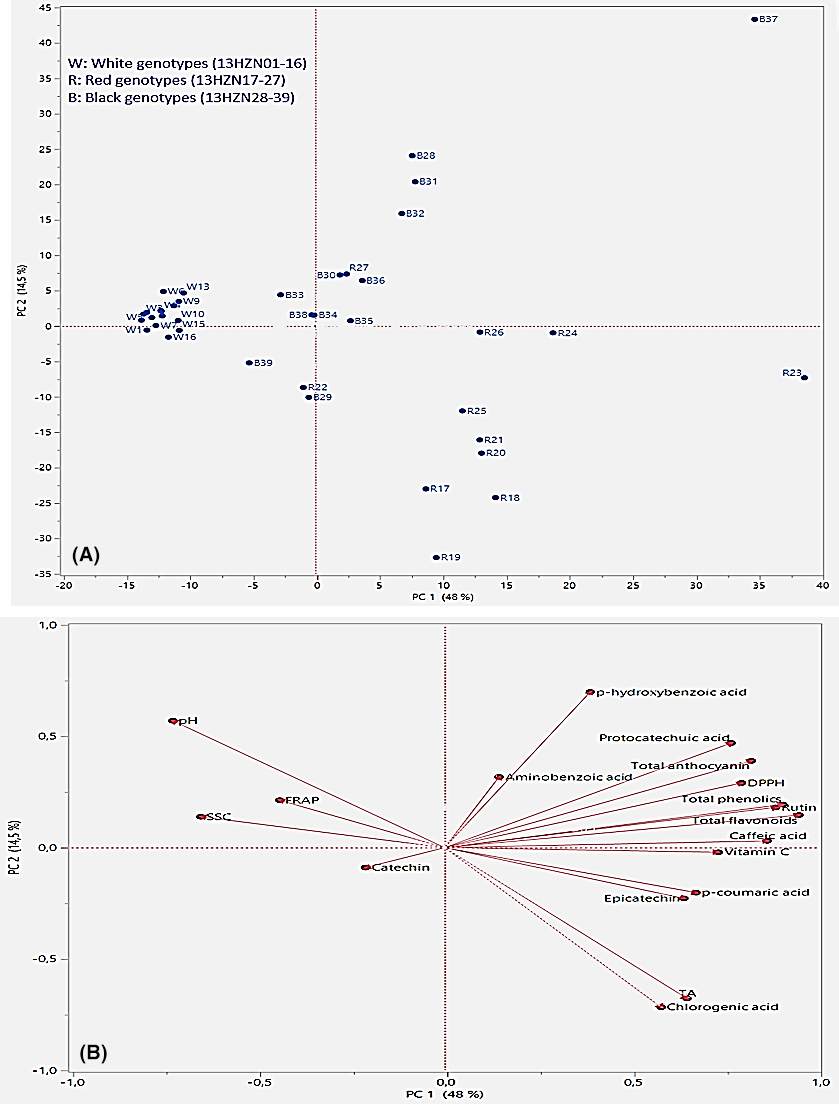
Principal component analysis (PCA).

Principal component analysis (PCA) was performed to determine the relationships between the examined biochemical traits and genotypes. Bartlett's sphericity test results showed that the correlations between variables were large enough for PCA (*p* < .0001) in Table 6. In PCA analysis, the components with attribute (eigen) values more than 1.00 are considered as “significant” (Alpar, 2011). According to the attribute (eigen) values, it was determined that the first four principal components considered to be important explained 82.12% of the total variance and the variance explanation rates of these principal components were 47.98% [(PC1 (first principal component)), 14.41% (PC2 (second principal component)), 12.47% (PC3 (third principal component)), and 7.24% (fourth principal component (PC4)]. Total flavonoid (0.32), total phenolic compound (0.30), rutin (0.30), caffeic acid (0.29), total anthocyanin (0.27), DPPH (0.27), protocatechuic acid (0.26), and vitamin C (0.24) are the most effective positive variables on PC1. pH (−0.24), SSC (−0.22), and FRAP (−0.15) are the most effective negative variables. p‐Hydroxybenzoic acid (0.43), pH (0.35), protocatechuic acid (0.29), and total anthocyanin (0.24) are the most effective positive variables on PC2, while chlorogenic acid (−0.44) is the most effective negative variable. On PC3, aminobenzoic acid (0.50), FRAP (0.40), catechin (0.38), and SSC (0.34) are the most effective positive variables, while p‐hydroxybenzoic acid (−0.18) and DPPH (−0.16) are the most effective negative variables. Epicatechin (0.41) and FRAP (0.37) are the most effective positive variables on PC4, and p‐hydroxybenzoic acid (0.30) and protocatechuic acid (−0.27) are the most effective negative variables in Table 6. PCA correlation plot in Figure 1a showed that the genotypes were divided into two main groups. White genotypes were separated from black and red genotypes, and separation occurred along PC1. The most effective variables responsible for the formation of the groups were determined using the load chart in Figure 1b. SSC, FRAP, and pH were the most important variables that distinguish white genotypes from black and red genotypes. Protocatechuic acid, p‐hydroxybenzoic acid, aminobenzoic acid, rutin, caffeic acid, total anthocyanin, DPPH, total phenolic, and total flavonoid were the most important variables that distinguish genotypes S28, S30, S31, S32, S36, S37, and K27 from other genotypes. Vitamin C, p‐coumaric acid, epicatechin, titratable acidity, and chlorogenic acid were the most important variables that distinguish genotypes K17, K18, K19, K20, K21, K23, and K25 from other genotypes. The mentioned black and red genotypes had a richer content than other genotypes in terms of variables that cause discrimination.

**TABLE 6:** Attribute (eigen) values of the first four principal components, the variation rates, and eigenvector values.

Number	1	2	3	4
Principal components (PCs)				
Attribute (eigen) values	8.63	2.59	2.24	1.30
Explained variation rate (%)	47.98	14.41	12.47	7.24
Cumulative rate of variation (%)	47.98	62.39	74.87	82.12
Chi‐square	2614.84	1724.84	1423.49	1045.95
Degrees of freedom	149.70	147.80	134.83	121.51
Chi‐square test significance level	<.0001*	<.0001*	<.0001*	<.0001*
Eigenvector values				
Total phenolics	0.30	0.11	0.02	0.21
Total flavonoids	0.32	0.09	0.04	0.16
DPPH	0.27	0.17	−0.16	0.21
FRAP	−0.15	0.13	0.40	0.37
Total anthocyanin	0.27	0.24	−0.00	0.22
Vitamin C	0.24	−0.01	−0.11	0.19
Aminobenzoic acid	0.04	0.19	0.50	−0.25
Protocatechuic acid	0.26	0.29	0.01	−0.27
Epicatechin	0.21	−0.14	0.23	0.41
p‐Coumaric acid	0.22	−0.12	0.27	−0.24
Rutin	0.30	0.11	0.17	−0.15
Caffeic acid	0.29	0.01	0.16	−0.03
p‐Hydroxybenzoic acid	0.13	0.43	−0.18	−0.30
Chlorogenic acid	0.19	−0.44	0.10	−0.14
Catechin	−0.07	−0.05	0.38	−0.25
pH	−0.24	0.35	0.16	0.05
SSC	−0.22	0.08	0.34	0.24
Titratable acidity	0.21	−0.41	0.07	−0.03

* Significant.

Genotypes S29, S39, and K22, which have negative values for both PC1 and PC2, were characterized by low catechin content. Protocatechuic acid, total anthocyanin, total phenolic, rutin, and total flavonoid can be said to be the variables with the most significant impact on DPPH based on their proximity in the load plot in Figure 1b. In general, the black and red genotypes have been found to be more nutritionally interesting than white genotypes. It was observed that there was a wide variation between both species and genotypes in terms of the biochemical properties examined, and the variation between genotypes was lower in white mulberries and higher in black and red mulberries in Figure 1a.

### 3.6 Clustering analysis

**FIGURE 2:**
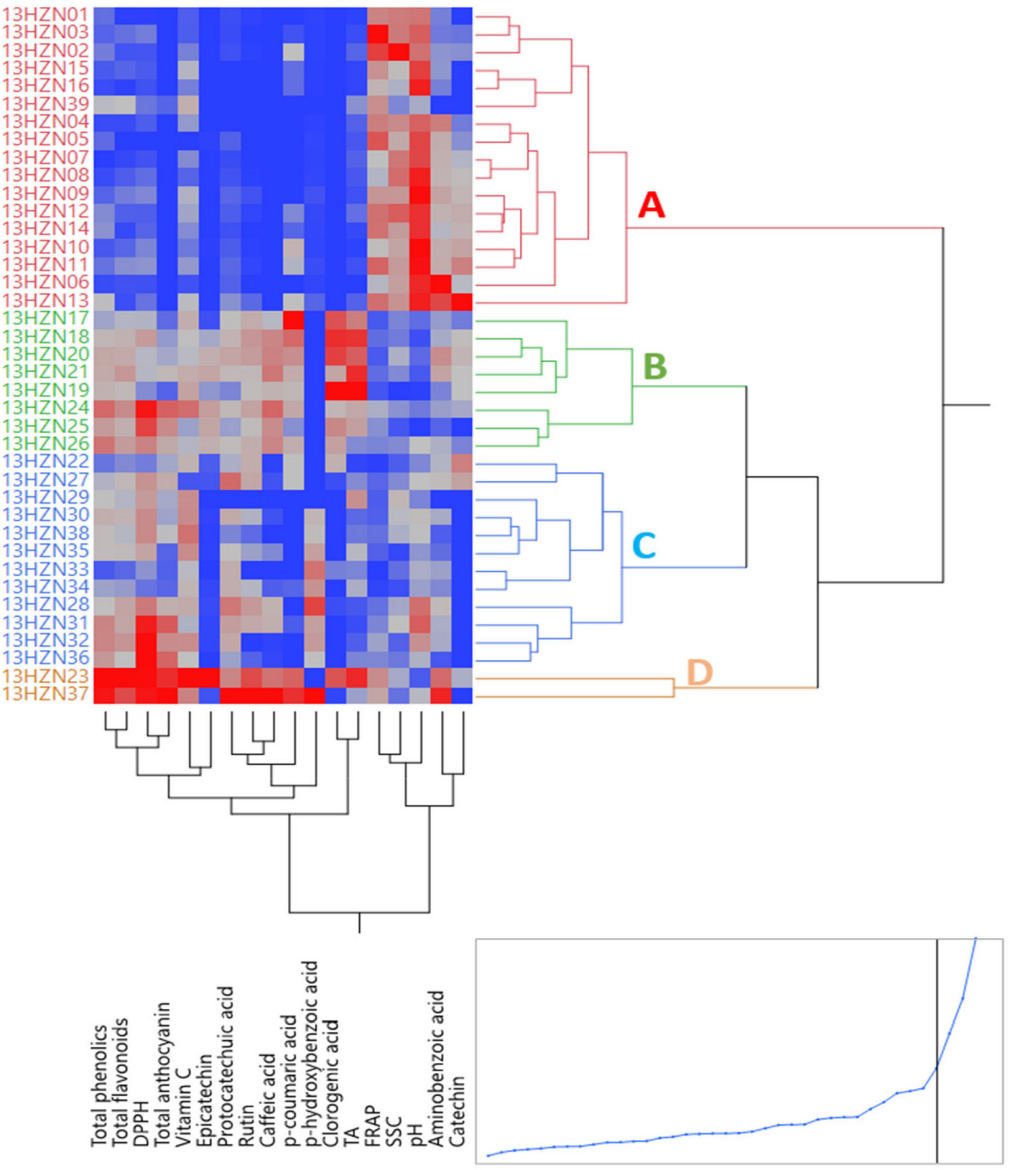
Clustering analysis.

**FIGURE 3:**
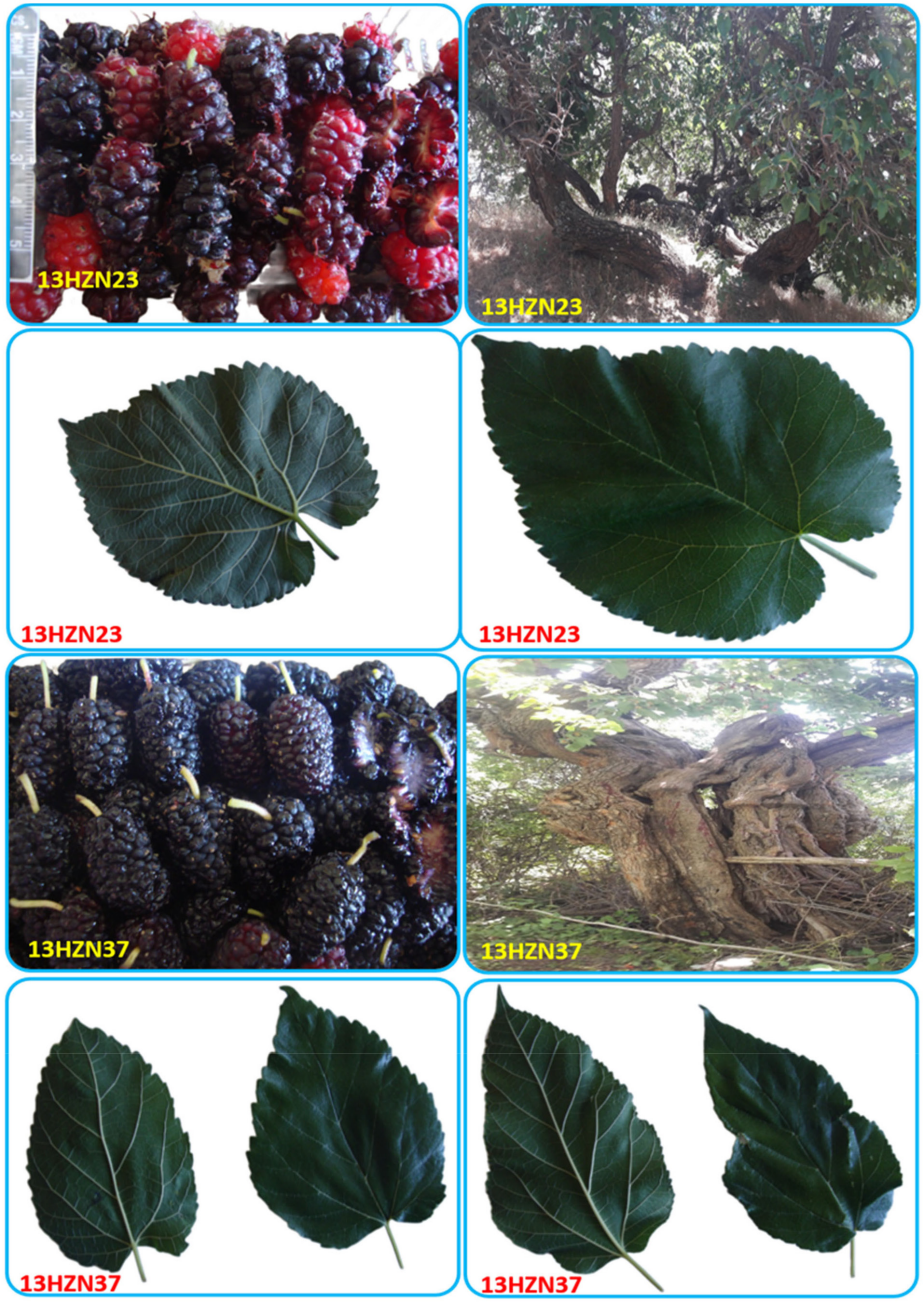
Genotypes (13HZN23 and 13HZN37) with superior characteristics.

Clustering analysis was performed to determine the similarities and differences between genotypes in terms of the biochemical characteristics examined. According to the clustering analysis results, the genotypes were divided into four groups in Figure 2. Sixteen white and one black genotype (13HZN39) in group A had the highest FRAP, catechin, pH, and TSS values and the lowest values of total phenolic compound, total flavonoid, DPPH, total anthocyanin, vitamin C, protocatechuic acid, and epicatechin, compared to the genotypes in other groups. They showed the values of rutin, p‐hydroxybenzoic acid, and titratable acid. The eight red genotypes in group B had the highest chlorogenic acid and titratable acidity values and the lowest pH and SSC values compared to the genotypes in the other groups. The genotypes in this group showed the highest values after the genotypes in group D in terms of total phenolic, total flavonoid, epicatechin, p‐coumaric acid, rutin, caffeic acid, and FRAP values. Two red (13HZN22 and 27) and 10 black genotypes in group C showed the lowest FRAP, aminobenzoic acid, p‐coumaric acid, and chlorogenic acid values compared to the genotypes in the other groups. The genotypes in this group had the highest values after the genotypes in group D in terms of DPPH, total anthocyanidin, vitamin C, protocatechuic acid, and p‐hydroxybenzoic acid values. Genotypes 13HZH23 and 13HZN37 in group D had the highest total phenolic compound, total flavonoid, DPPH, total anthocyanin, vitamin C, aminobenzoic acid, protocatechuic acid, epicatechin, p‐coumaric acid, rutin, caffeic acid, and p‐hydroxybenzoic acid compared to the genotypes in other groups. These were the genotypes with the lowest acid values and catechin amount in Table 7. It was observed that the most different genotypes in the dendogram were genotypes 13HZH23 and 13HZN37, which showed the highest values in terms of the 12 biochemical properties examined. These genotypes can be used in breeding studies to improve the biochemical properties of mulberry fruit and as food supplements in diet programs, as well as for clonal propagation and development of commercial varieties (Figure 3).

**TABLE 7:** Average discrimination values of groups according to the biochemical characteristics examined.

Group	A	B	C	D
Number	17	8	12	2
Total phenolics^a^	1.41 ± 0.85 c	5.19 ± 1.73 b	3.86 ± 1.81 b	11.15 ± 0.23 a
Total flavonoids^b^	0.63 ± 0.41 c	2.90 ± 0.77 b	2.12 ± 0.89 b	6.99 ± 1.18 a
DPPH^c^	0.81 ± 0.35 c	3.14 ± 1.48 b	3.45 ± 1.64 b	5.62 ± 0.07 a
FRAP^c^	25.75 ± 8.01 a	7.13 ± 3.17 b	5.88 ± 2.86 b	23.10 ± 3.18 a
Total anthocyanin^d^	0.03 ± 0.12 c	3.02 ± 2.61 b	3.07 ± 2.72 b	10.89 ± 0.98 a
Vitamin C^e^	10.18 ± 3.15 c	16.52 ± 4.02 b	17.30 ± 4.89 b	27.74 ± 7.61 a
Aminobenzoic acid^f^	0.53 ± 0.35 b	0.39 ± 0.22 b	0.27 ± 0.16 b	1.13 ± 0.22 a
Protocatechuic acid^f^	0.10 ± 0.10 c	0.84 ± 0.32 b	1.12 ± 0.74 b	2.98 ± 1.36 a
Epicatechin^f^	0.02 ± 0.45 c	15.50 ± 11.14 b	0.31 ± 0.34 c	50.23 ± 70.01 a
p‐Coumaric acid^f^	0.05 ± 0.07 c	0.42 ± 0.29 b	0.03 ± 0.06 c	0.78 ± 0.14 a
Rutin^f^	0.25 ± 0.51 d	14.19 ± 4.31 b	7.26 ± 7.11 c	44.59 ± 8.59 a
Caffeic acid^f^	−3.61 ± 0.00 c	30.56 ± 11.65 b	3.88 ± 6.65 c	54.99 ± 20.76 a
p‐Hydroxybenzoic acid^f^	0.83 ± 0.89 c	1.78 ± 0.00 c	37.28 ± 33.58 b	70.05 ± 99.07 a
Chlorogenic acid^f^	−1.81 ± 0.00 c	34.54 ± 16.80 a	1.70 ± 4.31 c	20.99 ± 29.10 b
Catechin^f^	0.28 ± 0.38 a	0.16 ± 0.10 a	0.10 ± 0.25 a	0.08 ± 0.11 a
pH	6.35 ± 0.35 a	4.25 ± 0.57 c	5.36 ± 0.55 b	4.73 ± 0.93 c
SSC	26.32 ± 5.22 a	15.24 ± 2.35 b	16.23 ± 3.50 b	16.36 ± 2.82 b
Titratable acidity	0.22 ± 0.06 b	1.50 ± 0.69 a	0.40 ± 0.27 b	1.32 ± 1.20 a

*Note*: Means in columns with the same letter do not differ according to Duncan's test at *p* < .05.

^**a**^ g GAE kg^−1^ FW.

^**b**^ g QE kg^−1^ FW.

^**c**^ mmol TE kg^−1^ FW.

^**d**^ g cyanidin 3‐glucoside kg^−1^ FW.

^**e**^ mg 100 g^−1^.

^**f**^ mg kg^−1^, GAE, QE, and TE.

## 4 DISCUSSION

Fruit size, which is an important criterion for both fresh and dry consumption and processing of mulberries, varies depending on the species and the ecological factors of the region where it grows. The study showed that there were significant differences between species and genotypes in terms of fruit size. In terms of fruit weight, the black mulberry fruit were heavier, followed by red and white mulberry fruit. So much so that in black mulberry, the highest fruit weight value was 5.84 g (13HZN28), while in red and white mulberry, there were genotypes with 4.18 g (13HZN26) and 3.10 g (13HZN05), respectively. Gecer et al. (2016) studied black and white mulberries in Igdır province, and obtained similar results to those of our study in terms of fruit weight (4.15 g), while in many studies (Balik et al., 2019; Gundogdu et al., 2012; Keskin, 2016; Polat, 2013; Yilmaz et al., 2012) the lower fruit weight values were measured than our results. On the other hand, Gunes and Cekic (2003), Islam et al. (2006), and Erdem (2015) reported that the bigger fruit were obtained, in Tokat (4.37 g), Giresun (4.38 g), and Ordu (5.07 g), respectively. It is thought that the genetic differences between genotypes, different ecological characteristics of the regions, and the cultural processes applied are effective in the emergence of different results in the studies.

Biochemical properties of fruits, such as SSC, pH, titratable acidity, and vitamin C, significantly affect fruit quality and therefore consumer preferences. SSC and acidity levels, which are used as criteria to determine the maturity level of the fruit, are at the ideal level for consumption at the ripeness stage. The biochemical properties varied depending on the species and genotype. The SSC was found to be between 19.00% and 38.86% in white mulberry, while it was found to be between 11.86% and 21.90% and 12.60% and 18.90% in black and red mulberry, respectively. The titratable acidity rate was lower in white mulberry, but the highest acidity rate was detected in red mulberry. The significant differences in titratable acidity occurred between genotypes within the species. The highest pH value in the fruit was detected in white mulberry, followed by black and red mulberry. There were significant differences in the amount of vitamin C in the fruit depending on the species. There was a genotype with 33.13 mg 100 g^−1^ vitamin C content in red mulberry, but the highest vitamin C content was recorded as 24.10 mg 100 g^−1^ in black mulberry and 14.03 mg 100 g^−1^ in white mulberry, respectively. The chemical content of mulberry, such as SSC and titratable acidity, may vary depending on genetic, ecological factors and cultural practices. The fact that, the mulberry selection studies conducted in different regions have revealed that there were differences in terms of chemical content. For example, in Malatya, Elazığ, Erzincan, and Tunceli, SSC was 18.3%–28.3% (Aslan, 1998); in Van, SSC was 16.6%–19.2%, total acidity was 0.16%–0.26%, and pH was 6.2–7.4 (Cam, 2000); in Antalya, SSC was 15.6%–17.6%, total acidity was 1.94%–2.23%, and pH was 3.3–3.8 (Uzun & Bayir, 2009); in Tokat, SSC was 14.8%–17.5%, total acidity was 1.60%–2.11%, and pH was 3.3–5.7 (Gunes & Cekic, 2003); in Giresun, SSC was detected between 15.3% and 19.3%, total acidity 1.47%–2.17%, and pH 3.4–6.0 (Islam et al., 2006). When compared with these results, it can be seen that the SSC rate was relatively higher and the acidity rate was generally low in the genotypes examined in our study. In Malatya, Altun (2021) reported that SSC values in mulberry genotypes varied between 16.24% and 32.95%, that the harvest period was effective on SSC, and that SSC increased as the harvest progressed.

Fruit are considered a natural source of antioxidants such as anthocyanins and polyphenols, which may reduce the risk of cancer, heart disease, and stroke, prevent acute CNS damage (Gilgun‐Sherki et al., 2002), cardiovascular disease (Cuzzocrea et al., 2001), and asthma (Kirkham & Rahman, 2006). Previous studies have shown that the mulberry fruit exhibit various biological activities such as to protect the liver from damage, strengthen joints, facilitate urine output, lower blood pressure, act as laxative, hypoglycemic, expectorant, anthelmintic, odontalgic and emetic, antithrombotic (Yamamoto et al., 2006), antioxidant (Hassimotto et al., 2005; Naderi et al., 2004), antimicrobial (Takasugi et al., 1979) to anti‐inflammatory (Kim & Park, 2006) and neuroprotective (Kang et al., 2006) effects. These effects of mulberry fruit are due to their bioactive compounds, such as phenolics, flavonoids (Ercisli & Orhan, 2007), and anthocyanins (Lee et al., 2004). The content and concentration of bioactive compounds, such as vitamin C, minerals, phenolics, flavonoids, organic acids, and anthocyanins in fruit, may vary depending on the species, variety, and ecological conditions (Serra et al., 2011). In mulberry fruit, which are a species of fruit rich in phenolic compounds such as flavonoids, anthocyanins, and carotenoids, which have important effects on human nutrition and health, the content and concentration of these compounds vary depending on genetic and ecological factors (Munin & Edwards‐Lévy, 2011). In the study, total phenolic content varied depending on the species. The red mulberry fruit contained higher phenolic substances, which was followed by black and white mulberry. It was also determined that there were differences in phenolic substance content between genotypes within the species. It was observed that the total amount of flavonoids, which varied depending on the species and genotype within the species, was higher in red mulberry genotypes. Significant differences occurred in terms of antioxidant activity determined through DPPH and FRAP values, depending on the species and genotypes within the species. No significant amount of anthocyanins has been detected in white mulberry. In terms of anthocyanin amount, the highest and lowest values in red and black mulberry were at similar levels, and there were significant differences between genotypes in both species. Previous studies have shown that the bioactive compound content varied depending on the species (Akbulut et al., 2006; Aljane & Sdırı, 2016; Gecer et al., 2016; Kaya, 2020; Yoncaci, 2020) and that black mulberry had higher values in terms of content and concentration (Ercisli & Orhan, 2007; Jiang & Nie, 2015). However, it has been reported that the concentration of bioactive compounds varied among genotypes within the species (Gundogdu et al., 2017, 2018; Krishna et al., 2020; Yaman, 2021), and it has been suggested that geography (Khattak & Rahman, 2015) and altitude (Paunović et al., 2020) also affected the content and concentration of bioactive compounds. In the study, the phenolic compounds, such as aminobezoic acid, protocatechuic acid, epicatechin, p‐coumaric acid, rutin, caffeic acid, p‐hydroxybenzoic acid, chlorogenic acid, and catechin, were detected. The significant differences were detected in individual phenolic content in fruits according to the species and type of compound. Some bioactive compounds were not found in significant amounts in some genotypes. However, there were significant differences in quantity between genotypes. Balik et al. (2019), who reported that there were significant differences in phenolic compounds among mulberry genotypes, suggested that the most abundant individual phenolic compounds in mulberry were chlorogenic acid, rutin, gallic acid, protocatechuic acid, vanillic acid, ellagic acid, quercetin, catechin, caffeic, p‐coumaric, and ferulic acids, respectively. In similar studies, it was observed that different results were obtained regarding the phenolic compound that was found in the highest amount. So much so that Zadernowski et al. (2005) suggested that mulberry contains the most caffeic acid, while Radojković et al. (2012) listed the following in terms of quantity: gallic acid, chlorogenic acid, rutin, and quercetin. Gecer et al. (2016), Okatan (2018). and Gundogdu et al. (2017) suggested that the main phenolic compound in mulberry fruits is chlorogenic acid.

According to the results of PCAs, SSC, FRAP, and pH are the most important variables that distinguish white genotypes from black and red genotypes. Genotypes S28, S30, S31, S32, S36, S37 (13HZN28, 30, 31, 32, 36, and 37), and K27 (13HZN27) contain protocatechuic acid, p‐hydroxybenzoic acid, aminobenzoic acid, rutin, caffeic acid, and total anthocyanin. They have a richer content than other genotypes in terms of DPPH, total phenolics, and total flavonoids. Genotypes K17, K18, K19, K20, K21, K23, and K25 (13HZN18, 19, 20, 21, 23, and 25) are richer than other genotypes in terms of vitamin C, p‐coumaric acid, epicatechin, titratable acidity, and chlorogenic acid. According to the clustering analysis results, the genotypes were divided into four main groups. The genotypes 13HZH23 and 13HZN37 had the highest total phenolic compound, total flavonoid, DPPH, total anthocyanin, vitamin C, aminobenzoic acid, protocatechuic acid, epicatechin, p‐coumaric acid, rutin, caffeic acid, and p‐hydroxybenzoic acid compared to the genotypes in other groups. As a result of the study, it was observed that there was a wide variation among both species and genotypes in terms of the fruit and biochemical properties examined. It was determined that the variation between genotypes in terms of biochemical properties was lower in white mulberries but higher in black and red mulberries. It was concluded that genotypes 13HZH23 and 13HZN37, which showed the highest values in terms of the 12 biochemical properties examined, could be used as food supplements in breeding studies and diet programs to improve the biochemical properties of mulberry fruits, as well as for clonal propagation and development of commercial varieties.

## AUTHOR CONTRIBUTIONS

**Cuneyt Uyak:** Conceptualization (equal); data curation (equal); funding acquisition (equal); validation (equal). **Erdal Aglar:** Formal analysis (equal); methodology (equal); supervision (equal); writing – original draft (equal); writing – review and editing (equal). **Burhan Ozturk:** Methodology (equal); validation (equal); writing – original draft (equal); writing – review and editing (equal). **Adnan Doğan:** Conceptualization (equal); formal analysis (equal); investigation (equal); software (equal); supervision (equal); writing – original draft (equal). **Onur Tekin:** Formal analysis (equal); investigation (equal); methodology (equal); validation (equal).

## FUNDING INFORMATION

The study was supported by the project numbered FBA‐2022‐9867 of Yuzuncu Yil University Scientific Research Project Department.

## CONFLICT OF INTEREST STATEMENT

The authors declare no conflict of interest.

## Data Availability

N/A.
